# Defecation after magnesium supplementation enhances cognitive performance in triathletes

**DOI:** 10.1016/j.smhs.2024.04.001

**Published:** 2024-04-04

**Authors:** Chen-Chan Wei, M. Brennan Harris, Mengxin Ye, Andrew Nicholls, Ahmad Alkhatib, Luthfia Dewi, Chih-Yang Huang, Chia-Hua Kuo

**Affiliations:** a Laboratory of Exercise Biochemistry, University of Taipei, Taipei City, Taiwan, China; b Department of Kinesiology and Health Sciences, William and Mary, Williamsburg, VA, USA; c College of Physical Education and Science, Zhejiang Normal University, Jinhua, 321004, China; d College of Life Sciences, Birmingham City University, Birmingham, UK; e Department of Nutrition, Universitas Muhammadiyah Semarang, Semarang, Indonesia; f Cardiovascular and Mitochondrial Related Disease Research Center, Hualien Tzu Chi Hospital, Buddhist Tzu Chi Medical Foundation, Hualien, 970, Taiwan, China; g Center of General Education, Buddhist Tzu Chi Medical Foundation, Tzu Chi University of Science and Technology, Hualien, 970, Taiwan, China; h Department of Medical Research, China Medical University Hospital, China Medical University, Taichung, Taiwan, China; i School of Physical Education and Sports Science, Soochow University, Suzhou, China

**Keywords:** Stool empty, Constipation, Rectum, Exercise, Triathlon, Dantian

## Abstract

Constipation is correlated with diminished cognitive function, revealing a possible rectum-brain connection. In this counter-balanced crossover trial, 13 elite triathletes underwent a Stroop test to assess cognitive function and executive control. The Stroop test was conducted both with and without magnesium oxide intake, with a 1-week washout period between sessions. Oxygenation and blood distribution during the cognitive challenge were measured using Near-Infrared Spectroscopy (NIRS). Measurements were taken in both the prefrontal brain and the sub-navel region, where the highest glucose uptake was detected under the 18F-fluorodeoxyglucose Positron Emission Tomography (PET) scan. A significant reduction in completion time for the Stroop test was observed after defecation compared to the non-defecated condition (non-defecation: [27.1 ​± ​1.1] s; non-magnesium defecation: [24.4 ​± ​0.9] s; magnesium defecation: [23.4 ​± ​0.8] s, *p* ​< ​0.05). Stroop test performance was improved in all (100%, 13/13) of the participants after magnesium-induced defecation and most (69%, 9/13) of the participants after non-magnesium-induced defecation. While no alterations in oxygenation and blood distribution were observed in the prefrontal brain during the Stroop test, decreased oxygenation levels were observed in the sub-navel region under both defecated conditions, without significant changes in blood distribution (*p* ​< ​0.05). This data suggests an acute increase in oxygen consumption at this specific region. The result of this study suggests an unexplored causal link between the state of the rectum and cognitive performance. Magnesium supplementation to improved rectal emptying presents a novel application for optimizing cognitive function in athletes navigating intricate racing conditions.

## Abbreviation list

AMPA: α-Amino-3-hydroxy-5-methyl-4-isoxazolepropionic acid
3-D: 3-Dimensional
18F-FDG: 18F-fluorodeoxyglucose
GSK: Glycogen synthase kinase
NIRS: Near-infrared spectroscopy
PET: Positron emission tomography
PI3K: Phosphoinositide 3-kinase
*SE*: Standard error

## 1 Introduction

Participating in a triathlon competition requires mental exertion to navigate through unforeseen situations on the sports field, manage fatigue, and outperform a multitude of competitors. Numerous judgmental processes, involving rapid decision-making, are conventionally believed to take place within the central nervous system. Increased metabolic activity in the central nervous system inevitably demands more oxygen supply. This can be traced by increases in hemoglobin levels (combined oxy- and deoxy-hemoglobin) within the prefrontal regions of the brain during diverse cognitive tasks.^1^ We have previously reported a significant improvement in cycling performance and increased blood distribution to the prefrontal brain after defecation.^2^ However, the effect of defecation on cognitive burden during a judgmental task remains unexplored.

A significant correlation between constipation and poor frontal executive performance in patients with dementia suggests a possible communication between the rectum and the brain.^3^ However, the causal link between rectum and cognitive function has not been established by interventional studies. Recent paleontological evidence has presented a new argument suggesting that the enteric nervous system located in the abdomen should be considered the primary brain, distinct from the central nervous system that emerged in more recently advanced species.^4^ Nevertheless, the precise anatomical location responsible for rapid cognitive judgments within the abdominal region remains vaguely defined in humans.

In our preliminary work, we discovered a unique high glucose uptake region proximal to the rectum, exhibiting strong signal intensity comparable to that of the brain in whole-body 18F-fluorodeoxyglucose PET 3-D images. Therefore, tissue hemodynamic changes in both the sub-navel region and the prefrontal brain during a Stroop Color and Word Test were examined in real-time under both non-defecated and defecated conditions.^5^ The defecation treatment, used to minimize stress on the rectum, was conducted under conditions with and without magnesium supplementation. The Stroop Color and Word Test is designed to challenge the rapid cognitive components necessary for accurate decision-making through selective attention and mental judgment.^6^ Magnesium consumption is known to improve bowel movement frequency and stool consistency in those with functional constipation, leading to enhanced health-related quality of life.^7^^,^^8^ We hypothesize that there will be an improvement in the Stroop performance of young triathletes after rectal defecation using magnesium supplementation.

## 2 Methods

### 2.1 Participants

Thirteen college students (age ​= ​[20.3 ​± ​0.5] year [y], height ​= ​[167.0 ​± ​2.4] cm, body mass ​= ​[64.3 ​± ​2.7] kg) completed the defecation trials. To prevent potential dietary influence on defecation, same meals were provided by a dietitian (35–40 ​kcal⋅kg^−1^⋅day^−1^) one day before experiments. All participants were informed to avoid nutritional supplements and alcohol one week before the study.

### 2.2 Ethical approval

The study protocol was approved by University of Taipei Institutional Review Board (trial number: IRB-2019-087). All tests were performed according to the Helsinki Declaration for testing human participants. Descriptions of the testing procedure and potential risks involved in the study were provided to all the participants before receiving written and verbal consent.

### 2.3 Study design

A counter-balanced crossover repeated-measures, randomized trial was performed under two defecated (with or without magnesium oxide supplementation) and one non-defecated conditions. Participants visited the testing laboratory on three occasions separated by one week. All experimental procedures were performed at the same time of the day (06:00 a.m.–08:00 a.m.), and under similar laboratory environment (19 ​°C temperature, 60% relative humidity) after 10 ​hours (h) overnight fasting. All participants urinated in the morning right after sleep. To compensate the loss of fluid evaporation during sleep, water (300 ​mL) was provided in the morning before bowel evacuation. One week washout period between tests was used. All cognitive tests and hemodynamic measurements were performed exactly 1 ​h after the defecation (at 07:00 a.m.).

### 2.4 Stroop test

The Stroop Color-Word Test comprises five tasks designed to evaluate cognitive processing of color-word matches and mismatches.^5^ Participants complete tasks using five test cards, presented on an A4-sized laminated white paper on the table. Each card contains blocks of 50 words arranged in five evenly spaced columns, representing different colors.

Each participant was directed to verbally describe each of the five test cards as quickly and accurately as possible, following this sequence: Test card 1 (nominal): Participants read aloud 50 words spelled 'blue', 'green', or 'red' printed on the card in black ink. Task 2 (congruent phase): Participants read aloud 50 words printed in the correct color of red, blue, or green ink. Task 3 (congruent phase): Words were replaced by correctly colored boxes, and participants named the color. Task 4 (incongruent phase): Random words were written in either red, green, or blue inks (matching or mismatching), and participants correctly named the color. Task 5 (incongruent phase): Each color word was written in an incongruent color, requiring participants to correctly name the color (e.g., 'red' written in green ink). Throughout each test, participants were instructed by the assessor (referring to the answer sheet) to promptly rectify any errors before proceeding.

Time to complete the correct identification of items on each test card was measured. The number of errors made increases the time to complete each Stroop task and thus represents a decrease in cognitive performance. The durations (in seconds) to complete each test card were averaged to generate the final score of cognitive performance. All participants were assessed by the same assessor in a quiet room.

### 2.5 PET scan for NIRS detector positioning

The whole-body PET scan was a preliminary experiment aiming to identify metabolically active regions in the human body for further NIRS analysis during a cognitive performance assessment (Video 1). A 75-g glucose solution (500 ​mL) containing 18F-fluorodeoxyglucose (400 MBq) was orally ingested by a young participant aged 24 ​y. Multiple-frame step and shoot head-to-ankle PET imaging was started 1 ​h after oral glucose ingestion, using a PET scanner (SCANDITRONIX 4096 15WB Plus, General Electric, Milwaukee, WI, USA) of 97.5 ​mm axial FOV. The acquisition time for each step was 5 ​minutes (min). Transmission scan was performed with the same area of the emission scan. All image data were reconstructed by vendor-provided iterative reconstruction algorithm with correction of transmission scan. The reconstructed multi-frame images were then stacked together, decay-corrected to the start of the first scan step and converted to Mayo Clinic Analyze 7.5 format (Research Systems Inc., Boulder, CO, USA).

### 2.6 Hemodynamic measurements

NIRS optical detectors were placed on the prefrontal region (forehead above eyebrow) and lower abdomen region (2–3 inches below umbilicus where the brightest signal from the 18F-fluorodeoxyglucose PET scan image was located). PortaLite has a maximum detection distance of 4 ​cm depth into the targeted tissues.

The real-time changes of blood distribution in both sub-navel region and prefrontal brain were monitored by a non-invasive NIRS,^9^ using a two-detector probe 90 ​seconds (s) before the Stroop test. Blood distribution in both tissues was optically detected by measuring hemoglobin concentration (oxy- and deoxy-hemoglobin),^10^ while the oxygenation levels measured by relative oxygen saturation (%) were calculated as the oxyhemoglobin-to-total hemoglobin ratio during the same measuring period.^11^ Measures of total hemoglobin and oxygenation were collected at a frequency of 10 ​Hz via Bluetooth using Oxysoft software (Artinis Medical Systems, Elst, Netherlands). Both blood distribution and tissue oxygenation were measured using a wireless NIRS device (PortaLite, Artinis Medical System, Elst, Netherlands).

Participants were familiarized with the protocol and an experimenter corrected the procedure during a test trial a week before study. Given that mental and physical disturbances can influence NIRS values in the brain, experimenters meticulously ensured the participants were calm before establishing a baseline for each test condition. Participants were asked to relax completely until a stable baseline was established for 90 ​s before the Stroop test. The baseline is defined by minimal fluctuations in oxygenation (% oxygen saturation) and total hemoglobin without an obvious upward or downward trend for 90 ​s.

### 2.7 Statistical analysis

All data are expressed as mean ​± ​standard error (*SE*). A sample size of 5 participants was required to reach a power of 90% for the assessment of thinking performance on Stroop test between trials. A paired *t*-test was used to distinguish the magnitude of difference in time to completion for the Stroop test between defecated and non-defecated conditions as well as the hemodynamic changes against the baseline. Type 1 error less than 5% was considered significant. All analyses were performed using SPSS statistics software (version 25; IBM Corporation, USA).

## 3 Results

**Fig. 1:**
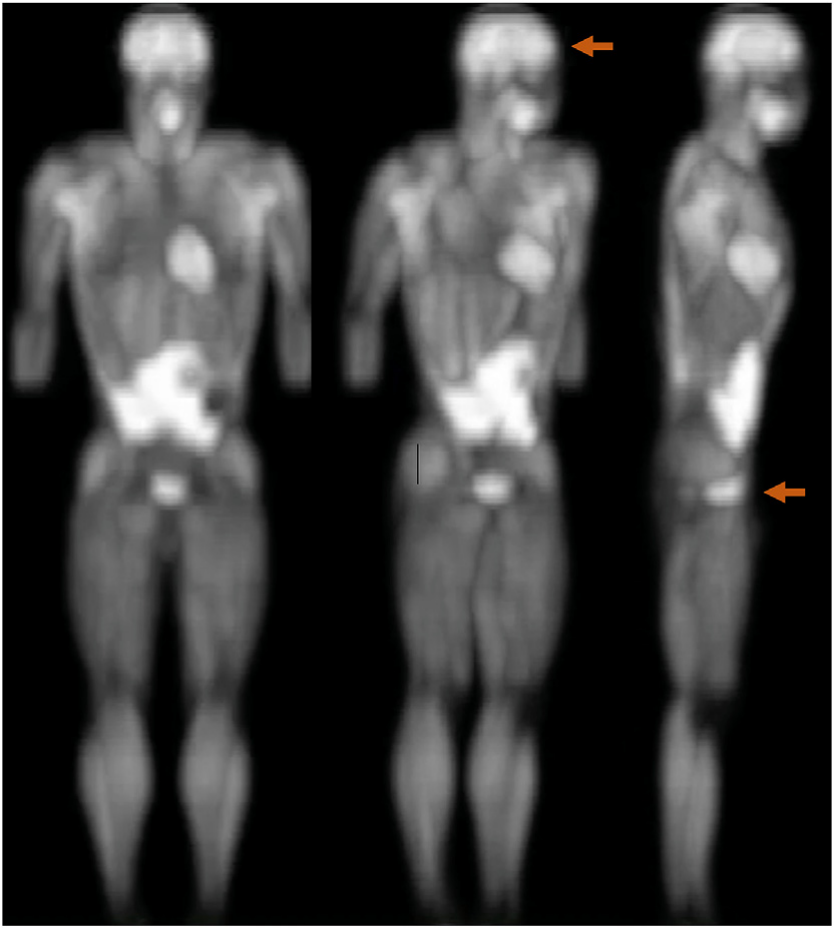
Detector positioning for near-infrared spectroscopy (NIRS) to prefrontal brain and sub-navel regions. Arrows indicate the position of NIRS detector probes placed for measuring real-time oxygenation and blood distribution in coronal view and sagittal view of the PET scan illustrated in Video 1.

Since glucose is the primary fuel for the nervous system, we used an 18F-fluorodeoxyglucose PET image to localize the regions with high glucose uptake for NIRS measurement. Fig. 1 presents the two-dimensional PET scan image taken 1 ​h after a 75-g of oral glucose ingestion (containing 18F-fluorodeoxyglucose). Excluding the region of glucose absorbing gastrointestinal tract in the upper abdominal region, the bright areas representing relatively high amounts of glucose uptake includes the prefrontal brain and sub-navel regions compared with the remaining parts of the body in a young man aged 24 ​y. A spherical shape within the sub-navel region (between navel and pubic areas) proximal to the rectum is clearly defined under the three-dimensional PET scan image (see the supplemental Video 1), physically separated from the glucose absorbing regions of the gut (stomach and small intestine). To examine the role of sub-navel and prefrontal brain regions in human cognition, detector probes of NIRS (covering tissues 4 ​cm in depth) were placed on both regions during a Stroop test, indicated by arrows in Fig. 1.

**Fig. 2:**
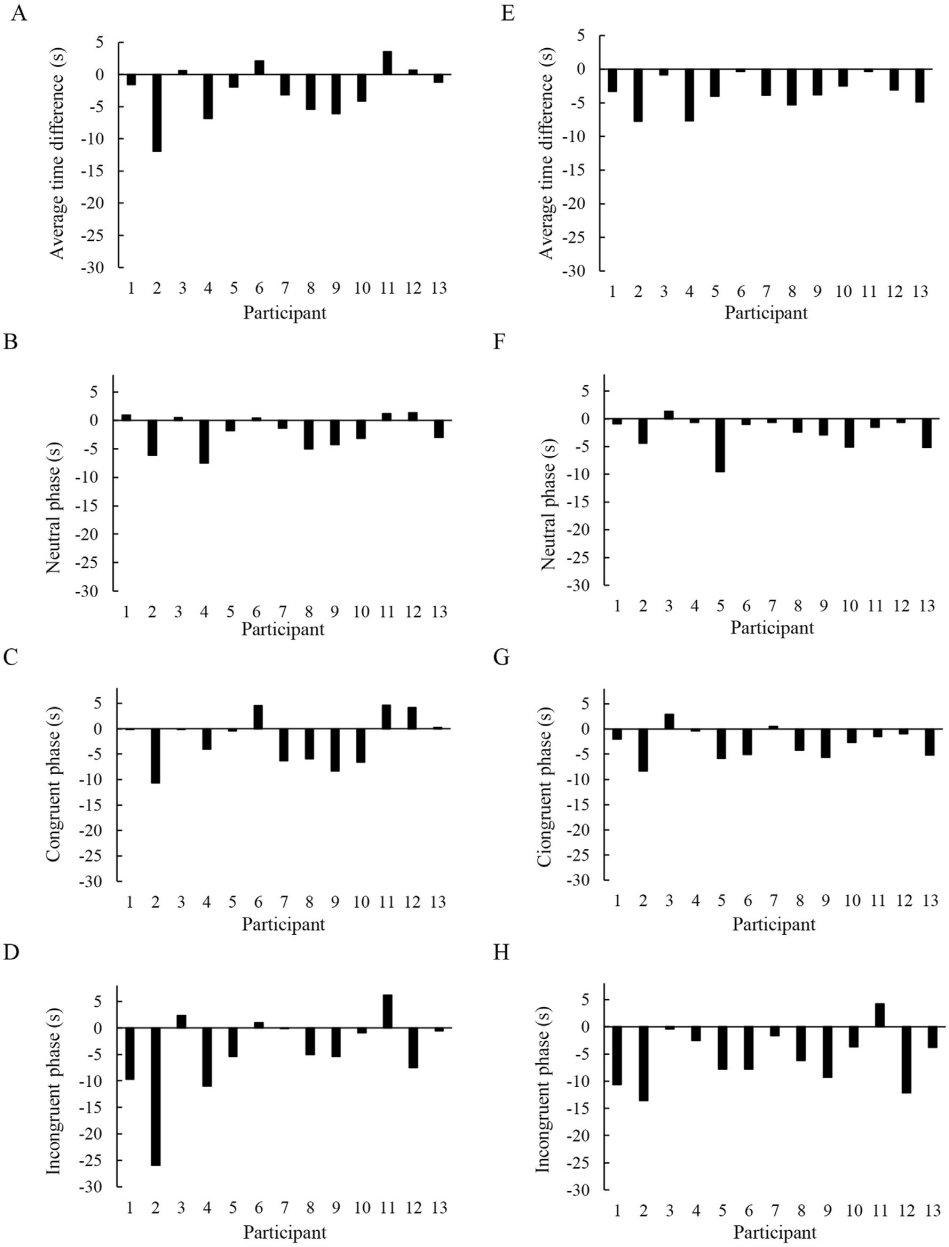
Defecation improved mental judgement (decreased time to complete the Stroop test). The number of errors made will increase the time to complete the Stroop test, and thus represent a decreased cognitive performance. The average time difference between non-defecated and defecated conditions was calculated by averaging the changes of performance on each of the 5 tasks within the Stroop test. Both voluntary defecation (A). Time difference during neutral (B), congruent (C), and incongruent (D) phases of the Stroop test is also illustrated in the non-magnesium defecated trial. Magnesium-defecation (E) decreased time to completion of the Stroop test without exceptions. Time difference during neutral (F), congruent (G), and incongruent (H) phases of the Stroop test is also illustrated in the magnesium defecated trial.

**Fig. 3:**
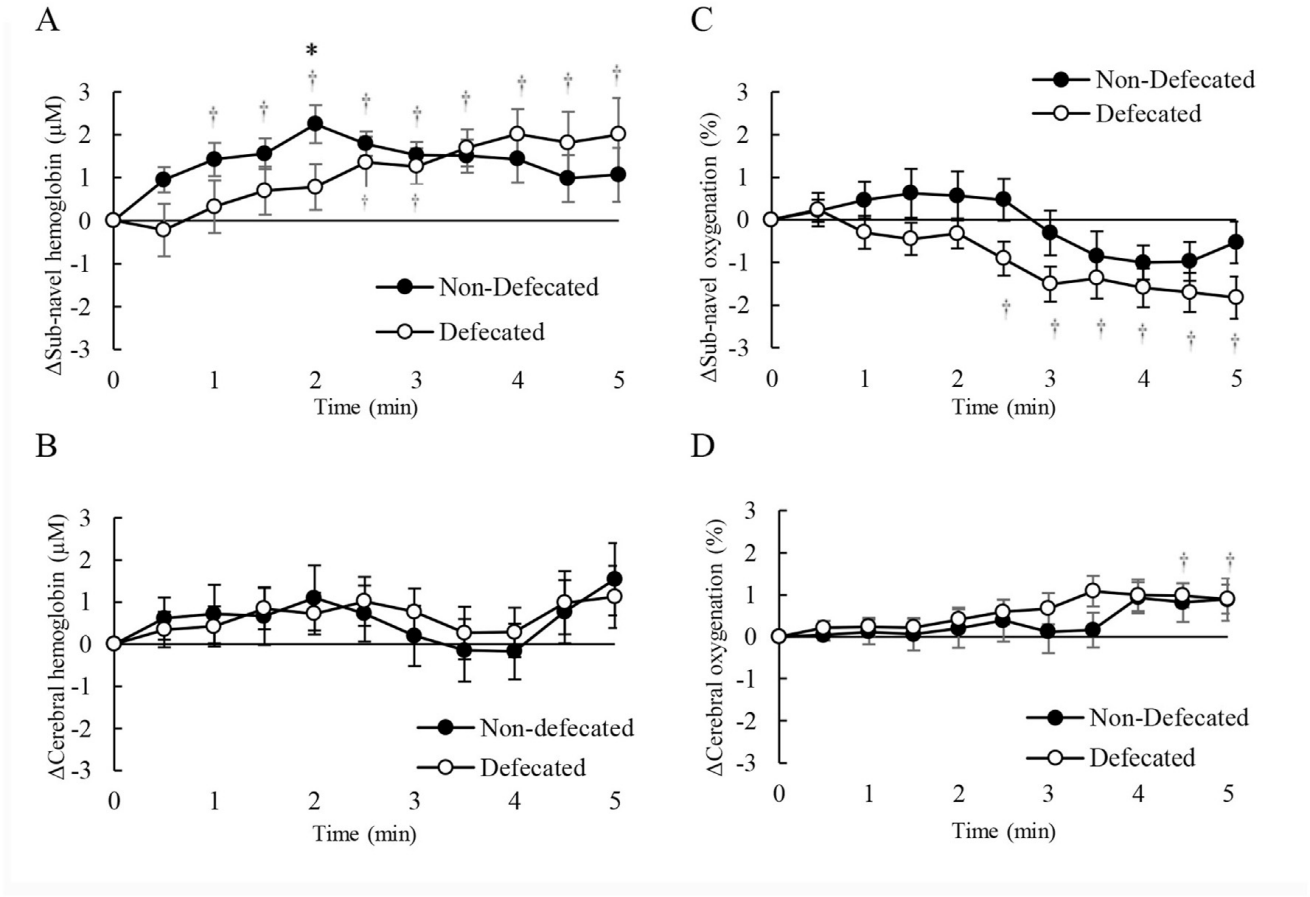
Stroop task increased oxygen consumption of sub-navel region during non-magnesium defecation. Tissue hemoglobin in the sub-navel region (A), reflecting blood distribution, was increased and about two-fold the amount of the prefrontal brain region (B). However, tissue oxygenation decreased in the sub-navel region (C) and was unchanged in the prefrontal brain region (D). † Significance against baseline, *p* ​< ​0.05. ∗ Significance against non-defecated condition, *p* ​< ​0.05.

**Fig. 4:**
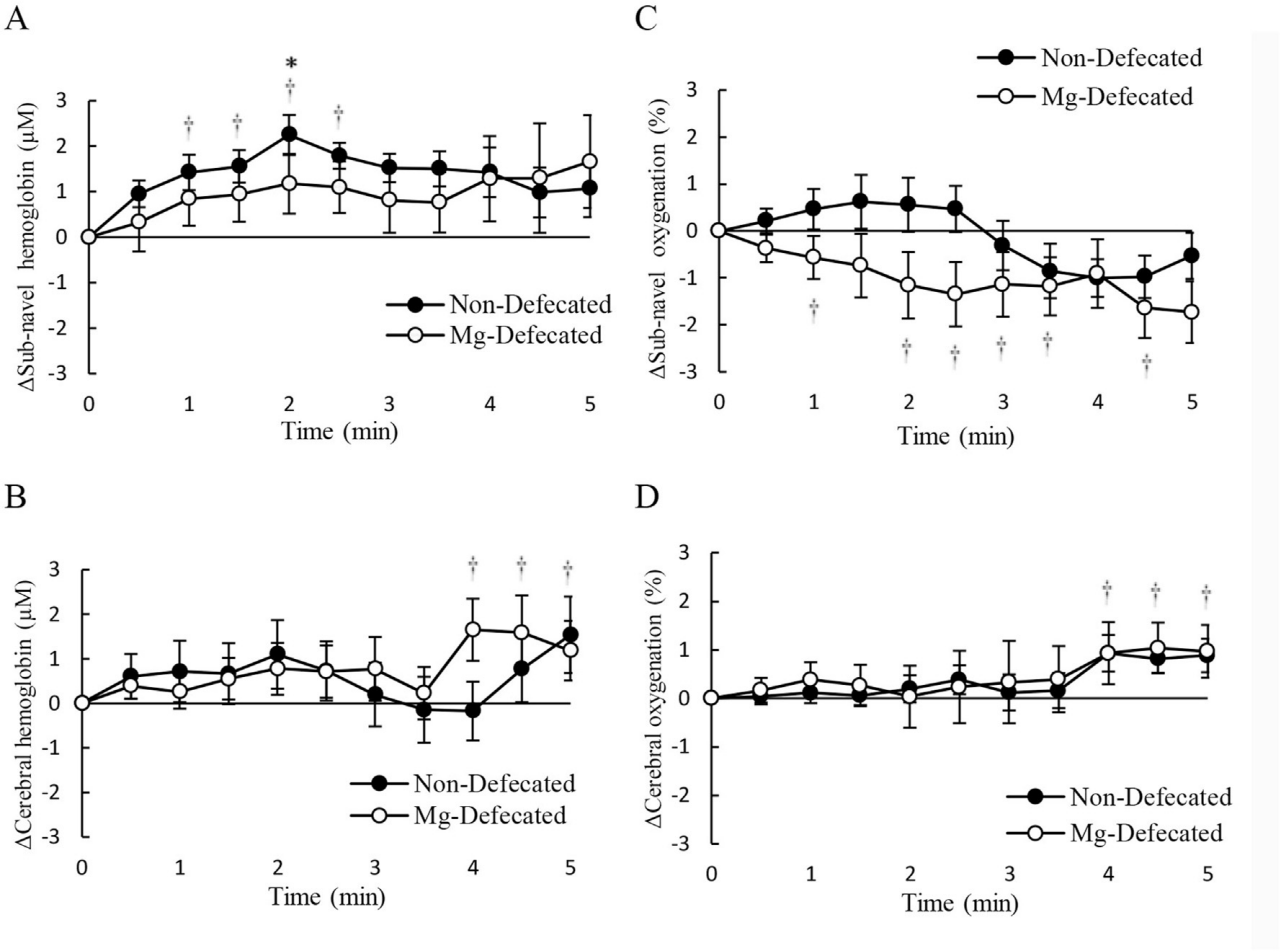
Oxygen consumption of sub-navel region increased during magnesium defecated condition during the Stroop task. Tissue hemoglobin, representing blood distribution, increased in the sub-navel region (A) and the prefrontal brain (B). However, tissue oxygenation decreased in the sub-navel region (C) and marginally increased in the prefrontal brain (D). † Significance against baseline, *p* ​< ​0.05. ∗ Significance against Non-defecated condition, *p* ​< ​0.05.

The Stroop test was used as a challenge of cognitive tasks requiring accurate mental judgements of the participants. Fig. 2 shows the difference in Stroop cognitive performance between defecated and non-defecated conditions assessed by time-to-completion for a Stroop test. Fig. 2A presents the time difference between non-magnesium defecated and non-defecated conditions. Fig. 2 B-D further shows the time difference during neutral, congruent, and incongruent phases of the Stroop test, respectively. Magnesium oxide was taken 12 ​h before the defecation. Fig. 2E presents the time difference between magnesium defecated and non-defecated conditions. Fig. 2 F–H further shows the time difference during neutral, congruent, and incongruent phases of the Stroop test, respectively. Tissue oxygenation (% oxyhemoglobin to total hemoglobin) and blood distribution (total hemoglobin) in the sub-navel and prefrontal cortex regions were measured during the Stroop test under the voluntary defecated condition (Fig. 3) and the magnesium defecated condition (Fig. 4). Decreased oxygenation without changes in blood distribution is evidence of increased oxygen consumption.

A negligible elevation in blood distribution (traced by total hemoglobin) to both sub-navel (Fig. 3A) and prefrontal brain (Fig. 3B) regions was observed during the Stroop test. A significant deoxygenation in the sub-navel region was observed during the incongruent phase (phase 4 and 5) of the Stroop test (Fig. 3C) and was more pronounced under the defecated condition. On the contrary, cerebral oxygenation was only negligibly elevated during the same period under both defecated and non-defecated conditions (Fig. 3D).

The effects of magnesium defecation on hemodynamic changes during the Stroop test are shown in Fig. 4. During the Stroop test, elevated blood distribution (total hemoglobin) for both sub-navel (Fig. 4A) and prefrontal brain (Fig. 4B) regions was similar for the non-magnesium defecated and magnesium defecated conditions. A significant sub-navel deoxygenation was observed during the Stroop test under the magnesium defecated condition (Fig. 4C). Apparently, this deoxygenation was not associated with reduced blood supply (total hemoglobin) (Fig. 4A). During the same testing period, the increases in cerebral blood distribution (Fig. 4B) and cerebral oxygenation (Fig. 4D) were minimal regardless of conditions.

## 4 Discussion

The novel finding of the present study is the substantial improvement in the Stroop test performance of triathletes following defecation, corresponding to a decreased level of oxygenation in the sub-navel region. This region, the lower abdomen, is notable for its high glucose uptake, as depicted in Video 1 using the 3-dimensional 18F-fluorodeoxyglucose PET scan. Despite the relatively smaller tissue volume, the signal intensity of 18F-fluorodeoxyglucose (which reflects the amount of glucose uptake in the tissues) in the sub-navel region was found to be comparable to that of the prefrontal brain. Additionally, we observed an acute increase in oxygen consumption during the Stroop test that was more pronounced in the sub-navel region compared to the prefrontal brain. This unexpected finding suggests a potential role of the enteric nervous system surrounding the rectum in immediate cognitive tasks which seems to correspond to the commonly described "gut feeling".

The most striking finding of this study is the unequivocal improvement observed in Stroop test performance for all participants consuming magnesium oxide. Magnesium oxide is widely used to soften feces and promote bowel movement.^12^ Even in the absence of magnesium oxide, defecation led to improved Stroop test results for 9 out of 13 individuals. This outcome suggests that magnesium by itself might independently influence the interaction between the rectum and the brain. This observation aligns with a previous study where magnesium consumption over a period of 6 weeks significantly alleviated depressive symptoms.^13^

In an animal study, the memory-enhancing effects of magnesium (both magnesium picolinate and magnesium oxide) were reported, mediated by AMPA-type glutamate receptors and the activation of the PI3K-Akt-GSK-3β signaling pathway.^14^ Many of these studies interpreted magnesium's beneficial impacts as being mediated primarily within the central nervous system.^15^ However, it's been documented that approximately 85% of ingested magnesium oxide remains in the rectal stool after ingestion,^16^ suggesting a confined biochemical influence beyond the gastrointestinal tract. Consequently, the findings from this current study present an alternative possibility that magnesium might directly influence cognitive function within the brain through its actions in the rectal region. More studies are required to elucidate the role of magnesium oxide in this process and its implications for athlete health and performance.

While both the sub-navel region and the prefrontal brain exhibited negligible increases in blood distribution during the administration of the Stroop test, declines in tissue oxygenation (reflected by the oxyhemoglobin to total hemoglobin ratio) were only evident within the sub-navel region, as opposed to the prefrontal brain. This outcome indicates an enhanced oxygen consumption within the sub-navel region during the Stroop test, thereby suggesting its responsiveness to swift cognitive tasks. This observation resonates with the hypothesis surrounding the enteric nervous system (localized in the gut), which is postulated to function as a rapid component when confronted with cognitive challenges. Remarkably, the rectal region harbors a substantial population of neurons and glial cells densely woven into the mesenteric walls.^17^ The mechanism to explain this unexplored link between the rectum and cognition remains elusive. Defecation can presumably lower the basal activity of the nerves in the rectal region and the corresponding sympathetic regions in central nervous system, allowing greater range of readjustment based on need. Alternatively, reduced autonomic burden may also spare energy for the parts of nervous system responsible for cognitive function.

In contrast, the emergence of the central nervous system during a later age of evolution on earth could conceivably be construed as constituting the brain's role in processing the demands of higher level and more intricate cognitive activities.^4^ The "first brain" proposition attributed to the enteric nervous system finds support in the presence of analogous systems within evolutionary precursors where central nervous systems were absent, and enteric nervous systems assumed the role early on.^4^ Collectively, the study's findings impart the notion that the enteric nervous system, particularly in proximity to the rectum, assumes a foundational function in managing cognitive needs.

The brain, defined as an organ of nervous tissue contained in the skulls of vertebrates, serves as the primary nexus for coordinating sensation, cognition, and motor function. However, recent studies, coupled with palaeobiological evidence, suggest that the conventionally anatomical confinement of nervous tissues within the skull may constrain our comprehension of cognitive processes. Remarkably, numerous species devoid of skulls exhibit adaptive and learning behaviors enabling survival in harsh environments, as exemplified by slime mold^18^ and jellyfish.^19^ The findings of acutely increasing oxygen consumption in the sub-navel region during the Stroop task prompt a reconsideration of including nervous (or non-nervous) tissue outside the skull as part of the “brain” or at least as part of cognitive function.

The sub-navel rectum region boasts an intricate neural network, establishing communication pathways with the central nervous system. As such, it holds promise as a potential locus for enhancing cognitive function. A noteworthy correlation between the severity of constipation and the onset of dementia underscores the rectum's influence on mental acuity in humans.^20^ The outcomes of this study establish a causal connection between the rectum and fast judgmental performance in the Stroop test. This discovery triggers a series of practical inquiries, encompassing whether variables like microbiota composition, satiety levels, and meal timing exert an impact on mental clarity. The impact of dietary restriction on cognitive capabilities has been extensively documented.^21^

Furthermore, the distinctly high glucose uptake observed in the sub-naval region during the 3-dimensional PET scan, as illustrated in the images of this study, intriguingly corresponds with anatomical sites known as "Dantian" in Chinese medical literature, "Hara" in Japan, and "Sacral Chakra" in India. These associations, spanning thousands of years, beckon further investigation. Currently, our understanding of this region's true significance in relation to mental performance remains limited by our reliance on ancient texts.

## 5 Conclusion

The findings of the study provide a novel perspective that underscores a connection between the rectum and cognitive performance in triathletes. Furthermore, defecation facilitated by magnesium supplementation emerges as a potent intervention capable of enhancing cognitive performance. The role of augmented rectal oxygen consumption during the cognitive test requires additional studies. Knowledge in this area could be improved by future studies that enlarge and diversify the participant pool to bolster the external validity of the results, given the current limitations stemming from a restricted number of elite triathletes.

## Ethical approval statement

The study protocol was approved by University of Taipei Institutional Review Board (trial number: IRB-2019-087). All tests were performed according to the Helsinki Declaration for testing human participants. Descriptions of the testing procedure and potential risks involved in the study were provided to all the participants before receiving written and verbal consent.

## Conflict of interest

**Chia-Hua Kuo** is an Editorial Board Member for Sports Medicine and Health Science and was not involved in the editorial review or the decision to publish this article. The authors declare that they have no competing interest.

## CRediT authorship contribution statement

**Chen-Chan Wei:** Writing – original draft, Software, Resources, Project administration, Methodology, Investigation, Funding acquisition, Formal analysis, Data curation, Conceptualization. **M. Brennan Harris:** Writing – review & editing, Validation, Conceptualization. **Mengxin Ye:** Writing – review & editing, Validation, Conceptualization. **Andrew Nicholls:** Writing – original draft, Validation, Methodology. **Ahmad Alkhatib:** Writing – review & editing, Validation. **Luthfia Dewi:** Writing – review & editing, Validation, Methodology. **Chih-Yang Huang:** Writing – review & editing, Validation, Conceptualization. **Chia-Hua Kuo:** Writing – review & editing, Project administration, Investigation, Conceptualization.
